# Using surveillance data to evaluate the effectiveness of inactivated/mRNA COVID-19 vaccine boosters in preventing fatal outcomes among severe COVID-19 cases during the current ambit of SARS-CoV-2 XBB and JN.1 variant circulation

**DOI:** 10.3389/fpubh.2025.1497399

**Published:** 2025-05-14

**Authors:** Eugene S. K. Lo, Lok Tung Wong, Serana C. Y. So, Kirran N. Mohammad, Ka Yi Law, Kam Suen Chan, Chung Lam Chan, Dawin Lo, Kin Hang Kung, Shuk Kwan Chuang

**Affiliations:** The Centre for Health Protection, The Department of Health, Kowloon, Hong Kong SAR, China

**Keywords:** COVID- 19, SARS-CoV-2, hospitalization, booster dose, mortality, inactivated vaccine, mRNA vaccine

## Abstract

**Background:**

Despite the rapid evolution of the SARS-CoV-2 viruses, vaccines targeting ancestral strains remain widely used. This study evaluates the effectiveness of ancestral strains inactivated and mRNA COVID-19 vaccine boosters in preventing fatal outcomes among severe COVID-19 cases during the circulation of the XBB and JN.1 variants.

**Methods:**

We analyzed 2,157 severe COVID-19 cases (aged ≥50) reported to the Centre for Health Protection from the hospital authority-managed public hospitals between January 30, 2023, and January 29, 2024. Logistic regression was used to investigate the relationship between vaccination status and fatal outcomes, adjusting for age, sex, and residential status in residential care homes for the older adult (RCHE), and other demographic factors.

**Results:**

Among the 2,157 cases, 764 (35.4%) succumbed within a 28-day follow-up. Fatal outcomes were more common among older individuals, RCHE residents, and those unvaccinated or with incomplete initial vaccination (zero to two doses). Fewer deaths had received ancestral strains mRNA or inactivated booster doses compared to those not receiving booster. Univariate logistic regression revealed the lowest in-hospital mortality odds ratio for mRNA booster recipients, followed by inactivated booster recipients, and then those with completed initial vaccination (three doses). After adjusting for confounders, booster vaccination remained significantly associated with reduced in-hospital mortality.

**Conclusion:**

Vaccines based on ancestral strains maintain some degree of effectiveness against recently emerged variants, offering insights for healthcare policies in regions where earlier generations of inactivated and mRNA vaccines continue to be administered.

## Graphical abstract

The visual abstract of the study.
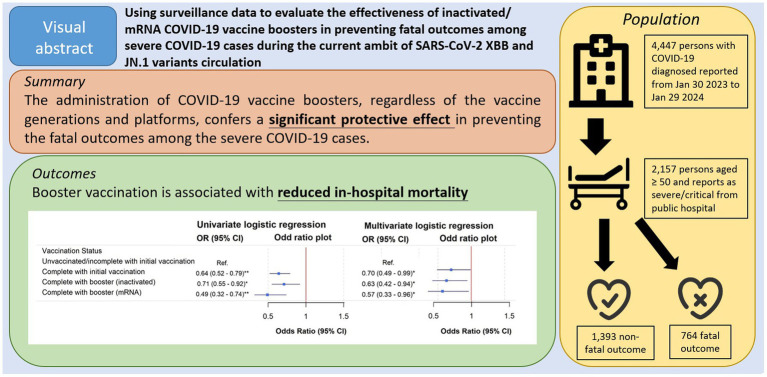

## Introduction

The COVID-19 outbreak spread globally after it emerged in 2020. Vaccination remains a crucial component in the prevention and control of the disease (1). In February 2021, the HKSAR Government set up a territory-wide vaccination initiative, the “COVID-19 Vaccination Programme” (The vaccination program), aimed at achieving herd immunity against COVID-19 and reducing the risk of severe clinical outcomes (2).

Under the vaccination program, both mRNA COVID-19 vaccines (3) and inactivated COVID-19 vaccines (4) were offered to members of the public. Two doses were recommended initially by the Scientific Committee on Vaccine Preventable Diseases and the Scientific Committee on Emerging and Zoonotic Diseases (Joint Scientific Committee (JSC)) (3). Later, the JSC further recommended that individuals aged 18 or older receive a third dose of either an mRNA or inactivated vaccine to enhance protection (2, 5). This was subsequently referred to as complete initial vaccination.

**Figure 1:**
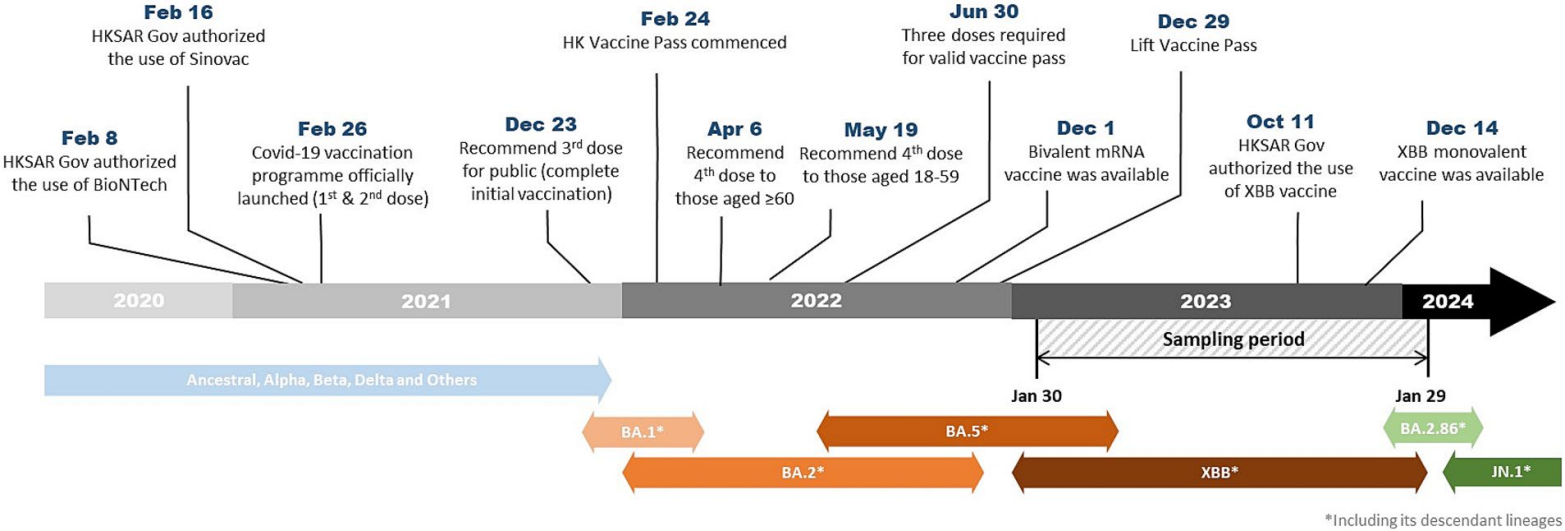
Timeline showing the evolution of predominant SARS-CoV-2 variants and key events of the vaccination program in Hong Kong.

In April and May 2022, a booster dose (fourth dose) was recommended to vulnerable groups (6) and also healthy adults (7) by the JSC, respectively. Figure 1 illustrates the evolution of predominant SARS-CoV-2 variants and key events of the vaccination program in Hong Kong.

The rapid evolution of the SARS-CoV-2 virus posed a significant challenge to public health efforts. As mutation speed outpaced vaccine development and administration, many ancestral strains vaccines were still in use. Many countries would require time and effort to utilize the existing supplies. This also raises questions about the sustained effectiveness of the vaccines currently in stock.

In addition to vaccination progress, the real-world performance of earlier-generation vaccines against rapidly emerging Omicron sublineages, such as XBB and JN.1, remains uncertain. While recent studies explored the effectiveness of the later monovalent Omicron XBB vaccines (8–10), there is limited research on the performance of ancestral strains inactivated and mRNA vaccines in the current epidemiological context. This knowledge gap is particularly relevant for regions where access to newer, updated vaccines may be limited, and understanding the effectiveness of available vaccines is crucial for informing public health strategies and vaccine deployment decisions.

Previous studies have explored the effectiveness of ancestral vaccines against earlier variants (11, 12). Evidence suggests that ancestral vaccines may still offer protection against newer variants (13–15). However, there are limited studies or evidence comparing the effectiveness of the inactivated vaccine to the mRNA vaccine. In Hong Kong, both inactivated and mRNA vaccines are available to the public. In this context, we aim to address the gap by evaluating the effectiveness of both vaccines under the same setting.

Our study uses surveillance data to evaluate the effectiveness of ancestral strains vaccines, including both inactivated vaccine and mRNA vaccine, in the prevention of fatal outcomes among severe COVID-19 cases in the Omicron-prevalent period. By focusing on hospitalized patients—a critical, high-risk segment of the population, we seek to provide insights into the performance of different vaccine types in the context of the current circulating variants.

## Methods

### Data source

COVID-19 has been one of the notifiable diseases in Hong Kong since 2020, and the Centre for Health Protection (CHP) of the Department of Health (DH) of Hong Kong maintains the COVID-19 cases database. The Hospital Authority (HA), a statutory body that provides over 90% of inpatient service in Hong Kong, maintains a comprehensive database of electronic health records from all public hospitals in the city. From 30 January 2023 onward, only severe and fatal COVID-19 cases are required to be reported by healthcare service providers (including the HA) to the CHP for surveillance purposes (16). The DH also maintains all vaccination records under the vaccination program. The COVID-19 database from the CHP, the electronic health records from HA, and the vaccination records from DH were mapped using a unique personal identifier of the patient. These population-based databases have been widely used in previous studies evaluating the effectiveness of vaccination strategies (17).

### Study design

**Figure 2:**
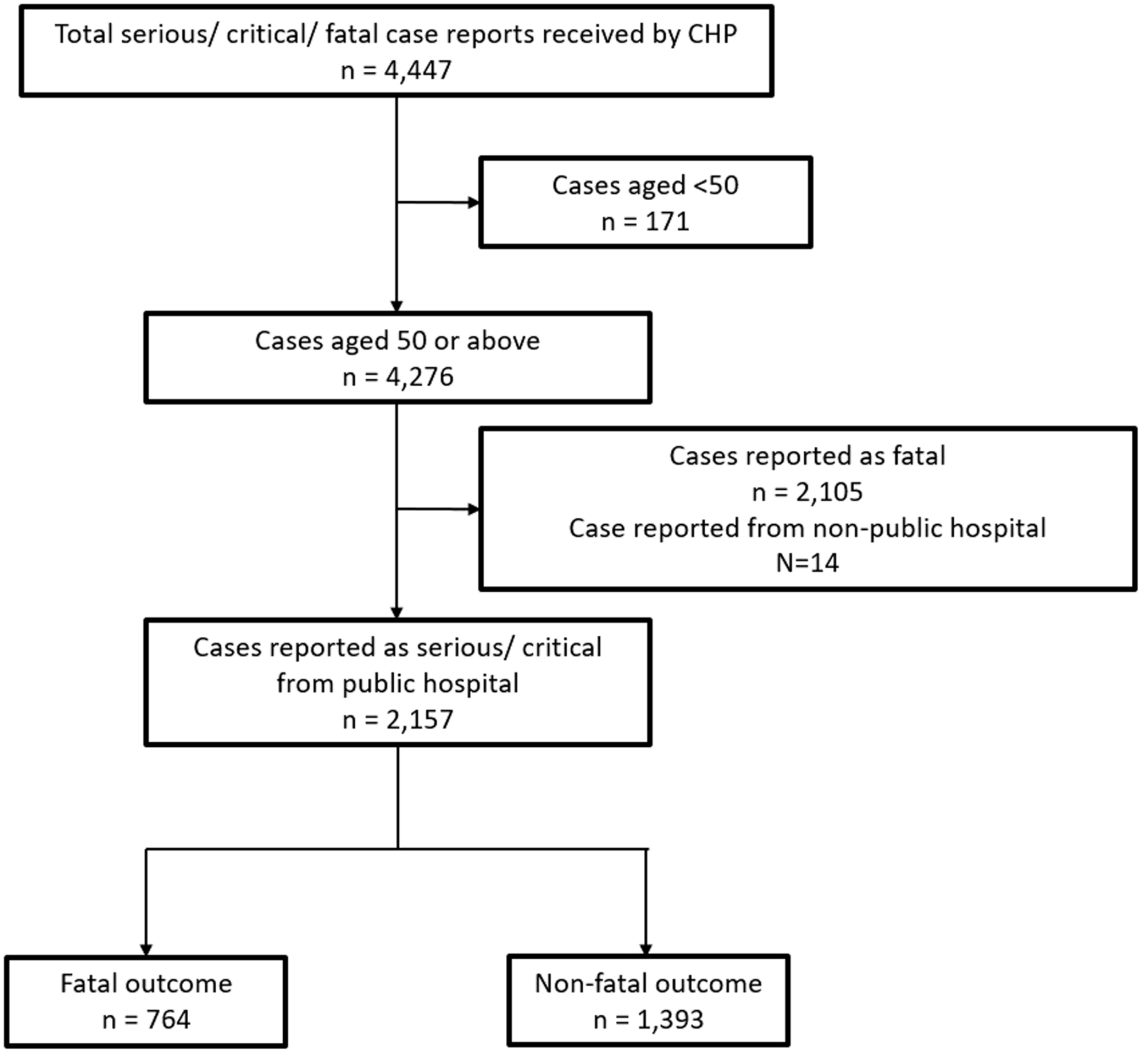
A flowchart of the study population.

Patients aged 50 or above with confirmed diagnosis of SAR-CoV-2 infection by either PCR or RAT and were in either serious or critical condition were included in the study. The inclusion period is from 30 January 2023 to 29 January 2024. Persons aged 50 or older are particularly susceptible to severe SARS-CoV-2 infections and are one of the high-risk priority groups to receive vaccines under the vaccination program in Hong Kong (18). Exclusion criteria included patients who were initially reported as fatal and patients reported from non-public hospitals (Figure 2).

The clinical criteria of a severe case include clinical manifestations such as severe pneumonia, sepsis, encephalopathy/encephalitis, myocarditis, multiple organ failure, shock, or other severe complications of COVID-19 that occur within 28 days of the first positive specimen collection date. Those cases requiring an oxygen supplement of 3–6 L/min are classified as serious, while those requiring intubation, extracorporeal membrane oxygenation, or high-flow oxygen with a flow rate >6 L/min are classified as critical.

Vaccination records from the DH include the vaccination type, date, and venue of dose administration for each individual vaccinated in Hong Kong. The case vaccination status was categorized as follows:

1. Unvaccinated/incomplete with initial vaccination (received zero or up to two doses of the COVID-19 vaccine);
2. Completed with initial vaccination (received three doses of the COVID-19 vaccine), which was recommended to the public by the JSC on 1 August 2022 (19);
3. Completed initial vaccination with inactivated vaccine booster (in addition to the initial three doses, received a valid dose of inactivated vaccine booster as the last received vaccine); and
4. Completed initial vaccination with mRNA vaccine booster (in addition to the initial three doses, received a valid dose of mRNA ancestral strains or mRNA bivalent (original/BA.4/5) booster as the last received vaccine).

It is assumed that a vaccination dose could only be effective from day 14 after injection, similar to other studies related to vaccine effectiveness (20, 21). Hence, if a severe or fatal case reports a vaccine injection within 14 days, the latest dose will be disregarded and not counted as a valid dose.

The DH has established an ethics committee to conduct ethical reviews of research projects carried out by the institutional staff and to provide advice on ethical matters related to research projects within its units (with the CHP being one of its functional arms). The DH has guidelines outlining the types of research projects that require ethical review and assessment by the committee. The committee’s secretary assessed our project and determined that the study met our institutional guidelines’ exemption criteria for ethics approval.

### Outcome

The outcome of the study was in-hospital mortality. Fatal outcomes were defined as those who died within 28 days of their first positive specimen collection date or first rapid antigen test (RAT) positive date (i.e., confirmed COVID-19 diagnosis), whichever is earlier.

### Statistical analysis

Age distribution between patients with and without fatal outcomes was compared using a two-sample *t*-test, while sex, chronic medical illness, residential care homes for the older adult (RCHE), and vaccination statuses were compared using a chi-squared test. We used univariate and multivariate logistic regression models to assess the relative in-hospital mortality among cases with different risk factor characteristics. Vaccination status was the primary study interest. Other adjusted confounders included age, sex, chronic medical illness (including a history of medical conditions such as hypertension, coronary heart disease, chronic heart failure, diabetes mellitus, chronic renal disease, and hyperlipidaemia), RCHE status, the interval between the event and the last valid dose, and the corresponding reporting month. The association between mortality and other potential risk factors, including age, sex, RCHE status, and time elapsed since the last valid dose, was also assessed using logistic regression. A simple sub-analysis was conducted to compare demographics and death status among subjects who received mRNA vaccine boosters based on different vaccine strains.

All analyses were performed using R Statistical Software (v4.2.2; R Core Team 2022) and Rstudio. The univariate and multivariate logistic regression models were created by the forestplot package (v3.1.6, Gordon and Lumley 2024) and gridExtra package (v2.3, Auguie 2017), with post-generation cutting and combining. A two-tailed *p*-value of <0.05 was considered statistically significant.

## Results

Of the 2,157 severe COVID-19 cases included in our study (Table 1), 764 cases had fatal outcomes. When compared with survived cases, fatal cases were older (83.4 ± 10.8 vs. 76.7 ± 11.2), had a higher proportion residing in RCHEs (25.9% vs. 15.3%) and being unvaccinated/or with incomplete vaccination (34.2% vs. 25.1%), and had a lower proportion for completed initial vaccination (43.7% vs. 49.9%) and boosters (22.1% vs. 25.0%). The sex ratio was comparable between the two groups. As for the subjects who were complete with an mRNA booster dose, 57 (44.9%) were receiving ancestral strains, and 70 (55.1%) were receiving a bivalent vaccine. No subjects in the study received valid XBB strain booster vaccine.

**Table 1:** Demographic characteristics between fatal cases and survived cases with severe/critical conditions (*n* = 2,157).

	Total (*n* = 2,157)	Fatal cases (*n* = 764)	Survived cases (*n* = 1,393)	*p*-value
**Age (mean ± SD)** ^**^	79.1 ± 11.5	83.4 ± 10.8	76.7 ± 11.2	<0.001
**Sex**				0.923
Female	809 (37.5%)	285 (37.3%)	524 (37.6%)	
Male	1,348 (62.5%)	479 (62.7%)	869 (62.4%)	
**Presence of chronic medical illness**	1873 (86.8%)	676 (88.5%)	1,197 (85.9%)	0.107
**Residing in RCHE** ^**^	411 (19.1%)	198 (25.9%)	213 (15.3%)	<0.001
**Vaccination status** ^**^				<0.001
Unvaccinated/ incomplete with initial vaccination	611 (28.3%)	261 (34.2%)	350 (25.1%)	
Completed with initial vaccination	1,029 (47.7%)	334 (43.7%)	695 (49.9%)	
Completed with booster (inactivated)	390 (18.1%)	135 (17.6%)	255 (18.3%)	
Completed with booster (mRNA)	127 (5.9%)	34 (4.5%)	93 (6.7%)	
**Time between event and last valid dose** ^*^				0.001
Unvaccinated	439 (20.4%)	187 (24.5%)	252 (18.1%)	
<180 days	351 (16.3%)	126 (16.5%)	225 (16.1%)	
≥180 days	1,367 (63.3%)	451 (59.0%)	916 (65.8%)	

See Supplementary Appendix 1 for the full table.

^*^p-value <0.05; ^**^p-value <0.001 were found between fatal cases and survived cases with severe/critical conditions.

**Figure 3:**
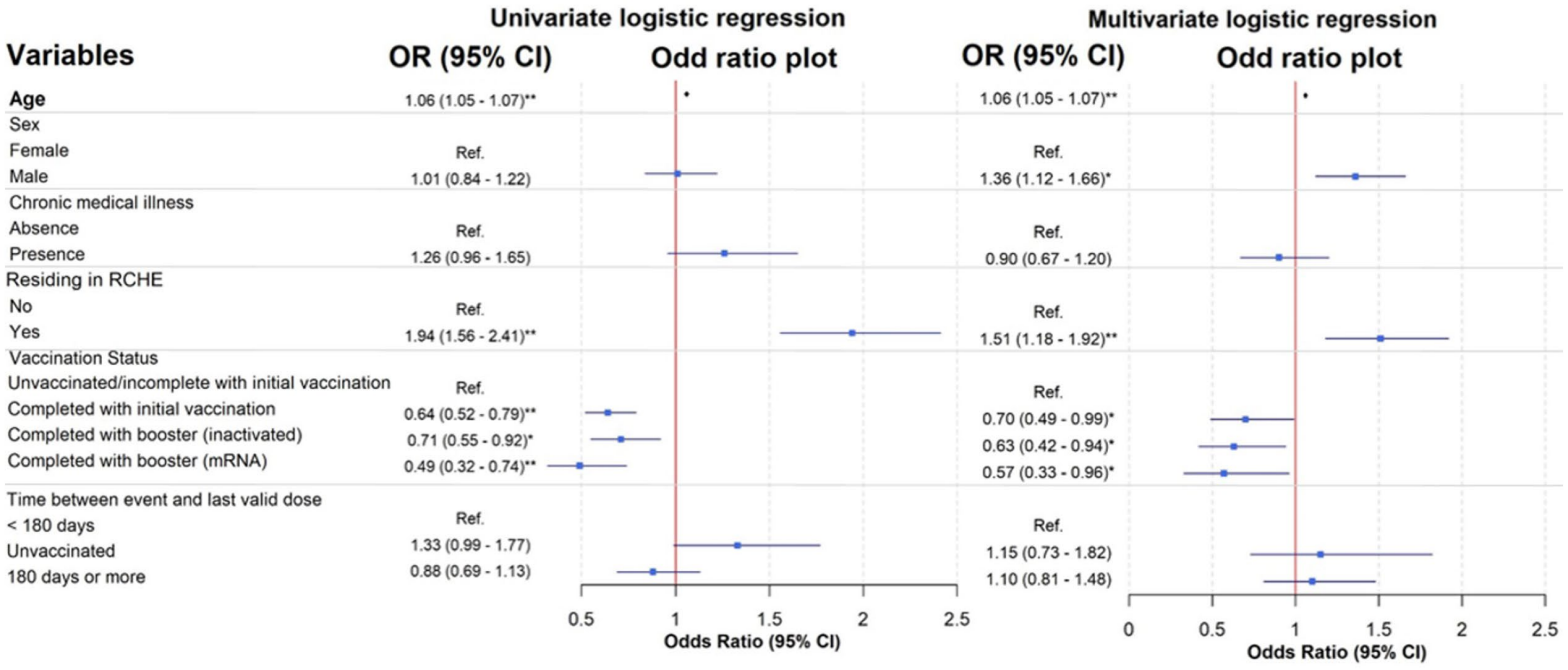
Univariate and multivariate logistic regression models of patients with different risk factors. See Supplementary Appendix 2 for a table of full analysis. ^*^p-value <0.05; ^**^ *p*-value <0.001 were found within the logistic regression model.

In the univariate analysis, when compared to the unvaccinated/incomplete with initial vaccination group, those who completed with initial vaccination [crude OR (95% CI): 0.64 (0.52–0.79)] or received either inactivated [crude OR (95% CI): 0.71 (0.55–0.92)] or mRNA vaccines [crude OR (95% CI): 0.49 (0.32–0.74)] as boosters in addition to the initial vaccination were associated with reduced mortality (Figure 3). After adjusting for the confounders, the association between vaccination status and reduced in-hospital mortality remained significant (OR (95% CI): completed with initial vaccination: 0.70 (0.49–0.99); inactivated booster: 0.63 (0.42–0.94); mRNA booster: 0.57 (0.33–0.96) compared to the unvaccinated/incomplete with initial vaccination group).

Moreover, the univariate analysis showed that older age [OR (95% CI): 1.06 (1.05–1.07)] and residence in RCHE [OR (95% CI): 1.94 (1.56–2.41)] were significant predictors of mortality. In the multivariate logistic regression model, significant predictors for mortality included older age (OR (95% CI): 1.06 (1.05–1.07), *p* < 0.001), being male (OR (95% CI): 1.36 (1.12–1.66), *p* = 0.002), and residing in RCHE (OR (95% CI): 1.51 (1.18–1.92), *p* < 0.001).

Subgroup-analysis: comparing the different generations of vaccines among mRNA booster recipients.

A simple *t*-test was conducted to compare the age of individuals who received mRNA vaccine boosters with the ancestral strains and those who received the bivalent strain. The results indicated no statistically significant difference between the two groups (*p* > 0.05). Furthermore, chi-square tests were conducted for other demographic variables. The test indicated that subjects who received the bivalent vaccine (64.3% within 180 days) as a booster were more likely to have a shorter interval between the event and the last valid dose compared to those who received the ancestral strains (12.3% within 180 days) (*p* < 0.001). Other variables, including sex, residential status at RCHE, chronic medical illness, and the corresponding reporting month, showed no statistical significance (*p* > 0.05). Similarly, death status did not significantly differ among the various strains of mRNA booster recipients. Nevertheless, this sub-group analysis may be underpowered due to small sample sizes (*N* = 127).

## Discussion

Our study utilizes real-world surveillance data to address a knowledge gap on the vaccine effectiveness of ancestral strains COVID-19 vaccine boosters against currently dominantly circulating XBB and JN.1 strains of SARS-CoV-2 virus. Both the ancestral strains inactivated and mRNA vaccines was protective against in-hospital mortality among the individuals who were admitted to the hospital due to SARS-CoV-2 infection during XBB and JN.1 predominant period. The one with initial vaccination and receiving booster had higher protection against those who were unvaccinated or without complete initial vaccination.

The circulating strain of COVID-19 in Hong Kong evolved during the study period. This period saw the decline of the BA.2 and BA.5 variants and the rise of the XBB variant and its sublineages, followed by the emergence of the JN.1 variant (22). The Public Health Laboratory Services Branch of the CHP analyzed 1,421 specimens (65.9%) from our studied cases for SARS-CoV-2 variants. We identified the majority (1,059, 74.5%) as XBB and its descendant lineages. We noted a small percentage (70, 4.9%) as BA.2.86 and its descendent lineages, while the remaining specimens (292, 20.6%) fell into other variants, such as BA.2 and its sublineage (96, 6.8%), BA.2.75 and its sublineage (142, 10.0%), and BA.5 and its sublineage (54, 3.8%). It is noteworthy that the BA.5 variant was circulating in the first half of 2023. The bivalent vaccine, available to the public in Hong Kong since December 2022, targets the Ancestral strain and the BA.4 and BA.5 strains (23). Nevertheless, in this study there are no subjects who received the bivalent mRNA vaccine (original & BA.4-5 strain) were infected with BA.4 or BA.5 strain.

Our findings indicate that age, sex, residential status in RCHE, and vaccination status are significant predictors of the likelihood of fatal outcomes within 28 days from the date of confirmed COVID-19 diagnosis for severe cases of COVID-19. The completion of the initial vaccination (three doses) appears to offer protection against mortality. The result is comparable to the findings from a previous study in Hong Kong in 2022, in which three doses of the COVID-19 vaccine provided the highest protection (24). Furthermore, an additional booster dose could provide even greater protection. Subjects who received the vaccine boosters, regardless of the ancestral strains inactivated vaccines, mRNA vaccine or the newer generations of mRNA vaccines, had a significantly lower likelihood of fatal outcomes than those who only completed the initial vaccination.

Our subgroup analysis findings indicate that there was no significant difference between subjects who received the bivalent mRNA vaccine as a booster and those who received the ancestral mRNA vaccine in terms of study outcomes and most demographic variables, including age, sex, and residential status. Given the gradual loss of protectiveness of COVID-19 vaccines due to waning, our study has demonstrated the importance and worthiness of periodically receiving a new booster dose (25–27), even of vaccines for ancestral strains. However, a relatively higher proportion of subjects who received the bivalent vaccine booster had less than 180 days between the event and the last valid dose. This observation is consistent with the fact that the bivalent vaccine was introduced toward the end of 2022 (28).

The vaccination coverage rate in Hong Kong is relatively high, even among high-income countries. According to Our World in data (29), the share of people who completed the initial COVID-19 vaccination protocol in Hong Kong is far higher than in average high-income countries (~15% difference). Sufficient vaccine supply and the vaccine pass policy would be the main drivers for the high vaccination rate in Hong Kong. The vaccine pass policy, implemented between February 2022 and December 2022 (30), encouraged the majority of the public to administer the vaccine, and the vaccination coverage boosted up quickly in that period (83% of the total population completed the third dose as of December 2022). As of that period, all available vaccines were ancestral strain-based, while vaccines targeting relatively newer variants were not available until October 2022. Many of the citizens completed their vaccination or received their booster vaccine with ancestral strains only.

Our results suggest that the ancestral strain-based vaccines retained a certain level of effectiveness against recently emerging variants. Although these strains demonstrate a certain extent of growth advantage and immune evasion capabilities based on the risk assessment of the WHO (31–33), vaccines developed from the ancestral strains still offer favorable and significant protection against fatal outcomes in severe cases.

The strength of this study is that it evaluates the association between the administration of ancestral strain-based vaccines, inactivated or mRNA vaccines, as boosters and COVID-19 case mortality under the current dominant circulation of the SARS-CoV-2 variants of interest. Our COVID-19 surveillance system collects case-based information on severe and fatal COVID-19 cases. We deployed the electronic medical records and vaccination records to ensure the accuracy and reliability of the data.

As the HA is the largest healthcare provider in the city, covering nearly all of our population, the majority of citizens from different socioeconomic backgrounds were included in the study, ensuring the generalizability of our results to hospitalized patients of all socioeconomic classes. Under the current COVID-19 vaccination strategies of Hong Kong, we can study and compare the effects of both inactivate and mRNA ancestral strain-based vaccines. Our findings reflect the effectiveness of COVID-19 vaccines in real-world settings, which provide reliable insights into the healthcare policies in countries or areas where previous generations of inactivated and mRNA vaccines are still deployed for administration. Our findings also support the WHO recommendations about the protection of receiving COVID-19 vaccines produced by whatever platforms under the current environment of SARS-CoV-2 variant circulations.

There are several limitations to our study. First, the study does not account for the potential confounding effects of a naturally acquired SARS-CoV-2 infection on vaccination. According to the previous recommendations of the JSC of the CHP (34, 35), naturally acquired infections could waive some of the vaccination requirements during the pandemic, affecting people’s incentive to receive the vaccine. Previous infection might also affect the severity of later infections and the relative effectiveness of the vaccines (36). Moreover, naturally acquired infection itself contributes to the hybrid immunity against COVID-19 among vaccinated individuals, and the inability to address it as a confounder raised uncertainties in the assessment of vaccine effectiveness. The temporal factor might be another potential cofounder that the study was unable to address, as the progress of the vaccination program has also coincided with the transition from BA.5 to XBB and JN.1 variants, which might vary in disease severity and immune escape from the vaccine.

Another limitation is the lack of case-based data on antiviral administration. Nevertheless, given that the prescription policy of antivirals for hospitalized severe COVID-19 patients in public hospitals is under the same clinical guidelines developed by the HA (37), we assume that all hospitalized patients under the HA had been prescribed antivirals. Last but not least, by excluding cases that were first reported to the CHP as fatal, our study tried to minimize the effect of antiviral drugs as a confounding factor in the study design.

The study evaluated fatal outcomes among severe hospitalized cases only. It did not address the effectiveness of booster vaccines in preventing non-fatal severe conditions or hospitalization as a result of the SARS-CoV-2 infection. Our findings, therefore, might not be able to indicate ancestral strains boosters’ role in reducing healthcare utilization or preventing non-fatal severe outcomes. A chi-square analysis of vaccination status and demographics between study subjects and the cases who first reported to the CHP as fatal (*n* = 2,105) was performed. Results (Supplementary Appendix 3) indicated that, compared to cases intially reported as deaths, our study subjects were younger, more likely to be male, and less likely to reside in RCHE. Vaccination status did not differ significantly. Further study is warranted.

Currently, we deploy patients with a confirmed COVID-19 diagnosis who died within 28 days of the first positive specimen collection date as the definition of fatal outcomes. However, in this study, over 80% of the included subjects had a pre-existing chronic illness. There is a lack of objective differentiation criteria to judge the degree of contribution of SARS-CoV-2 infection to the fatal outcomes (i.e., whether the case died of or died with COVID-19). Despite that, there was no statistically significant difference in the presence of pre-existing chronic illnesses among the groups. We assumed that this issue should not generate any directional bias in our evaluation.

The study did not capture the whole variant distribution among the subjects; only about 65% of the subjects have the available variant data. Consequently, conducting a specific variant sub-analysis was challenging due to the limited sample size.

The study provided limited insight into the effects of receiving multiple boosters. Given that only a small portion of subjects (~10%) had received multiple boosters before hospital admission, only the type of the last dose booster on record was considered for this study. Moreover, while our study provided valuable insights into the comparative effects of mRNA and inactivated vaccines as boosters, the relatively small sample size may have limited the statistical power to detect significant differences between the two vaccine types. Future studies with larger sample sizes would be beneficial to further validate these findings and provide more robust conclusions.

Finally, the study only includes severe COVID-19 cases aged 50 or above. Hence, the findings might not be generalizable to younger populations in the absence of supporting data.

## Conclusion

In conclusion, our findings demonstrated that the administration of COVID-19 vaccine boosters, regardless of the vaccine generations (ancestral or the newer bivalent for ancestral and BA.4/5) and platforms (inactivated or mRNA), confers a significant protective effect in preventing the fatal outcomes among the severe COVID-19 cases. The result aligns with the WHO’s endorsement of booster vaccines as a key measure in preventing the worst outcomes of COVID-19 (38). We supplement the information on the ancestral strains vaccine effectiveness under the current ambit of SARS-CoV-2 variant circulation.

## Data Availability

The original contributions presented in the study are included in the article/Supplementary material; further inquiries can be directed to the corresponding author.

## References

[1] World Health Organization. Statement on the fifteenth meeting of the IHR (2005) emergency committee on the COVID-19 pandemic. (2023). Available online at: https://www.who.int/news/item/05-05-2023-statement-on-the-fifteenth-meeting-of-the-international-health-regulations-(2005)-emergency-committee-regarding-the-coronavirus-disease-(COVID-19)-pandemic (Accessed December 30, 2024).

[2] Scientific Committee on Emerging and Zoonotic Diseases and Scientific Committee on Vaccine Preventable Diseases. Consensus interim recommendations on the use of Comirnaty vaccine in Hong Kong (as of 23 December 2021), (2022). Available online at: https://www.chp.gov.hk/files/pdf/consensus_interim_recommendations_on_the_use_of_comirnaty_vaccines_23dec.pdf (Accessed 20 August 2024).

[3] Scientific Committee on Emerging and Zoonotic Disease and Scientific Committee on Vaccine Preventable Diseases. Consensus interim recommendations on the use of COVID-19 vaccines in Hong Kong (as of Jan 7, 2021), (2021). Available online at: https://www.chp.gov.hk/files/pdf/consensus_interim_recommendations_on_the_use_of_covid19_vaccines_inhk.pdf (Accessed 20 August 2024).

[4] Scientific Committee on Emerging and Zoonotic Diseases and Scientific Committee on Vaccine Preventable Diseases. Consensus interim recommendations on the use of CoronaVac in Hong Kong (as of 19 February 2021) (updated on 19 November 2021), (2021). Available online at: https://www.chp.gov.hk/files/pdf/consensus_interim_recommendations_on_the_use_of_coronavac_in_hong_hong.pdf (Accessed 20 August 2024).

[5] Scientific Committee on Emerging and Zoonotic Diseases and Scientific Committee on Vaccine Preventable Diseases. Consensus interim recommendations on the use of COVID-19 vaccines in Hong Kong (as of 25 February 2022) (updated on 12 march 2022), (2022). Available online at: https://www.chp.gov.hk/files/pdf/consensus_interim_recommendations_on_the_use_of_covid19_vaccines_in_hong_kong_25feb_updated_on_12mar.pdf (Accessed 20 August 2024).

[6] Scientific Committee on Emerging and Zoonotic Diseases and Scientific Committee on Vaccine Preventable Diseases. Consensus interim recommendations on the use of COVID-19 vaccines in Hong Kong (as of 6 April 2022), (2022). Available online at: https://www.chp.gov.hk/files/pdf/consensus_interim_recommendations_on_the_use_of_covid19_vaccines_in_hong_kong_6_apr.pdf (Accessed 20 August 2024).

[7] Scientific Committee on Emerging and Zoonotic Disease and Scientific Committee on Vaccine Preventable Diseases. Consensus interim recommendations on the use of COVID-19 vaccines in Hong Kong (as of 19 May 2022), (2022). Available online at: https://www.chp.gov.hk/files/pdf/consensus_interim_recommendations_on_the_use_of_covid19_vaccines_in_hong_kong_19_may.pdf (Accessed 20 August 2024).

[8] Huiberts AJ Hoeve CE de Gier B Cremer J van der Veer B de Melker HE. Effectiveness of omicron XBB.1.5 vaccine against infection with SARS-CoV-2 omicron XBB and JN.1 variants, prospective cohort study, the Netherlands, October 2023 to January 2024. Eur Secur. (2024) 29:2400109. doi: 10.2807/1560-7917.ES.2024.29.10.2400109 PMC10986669 38456217

[9] Lee JA Jang H Ahn SM Seong JE Kim YK Sohn Y. Estimates of vaccine effectiveness of the updated monovalent XBB.1.5 COVID-19 vaccine against symptomatic SARS-CoV-2 infection, hospitalization, and receipt of oxygen therapy in South Korea - October 26 to December 31, 2023. Int J Infect Dis. (2024) 148:107249. doi: 10.1016/j.ijid.2024.107249, PMID: 39307179

[10] Wee LE Tang N Pang D Chiew C Yung C-F Chong CY. Effectiveness of monovalent mRNA vaccines against omicron XBB infection in Singaporean children younger than 5 years. JAMA Pediatr. (2023) 177:1324–31. doi: 10.1001/jamapediatrics.2023.4505, PMID: 37843856 PMC10580153

[11] Lopez Bernal J Andrews N Gower C Robertson C Stowe J Tessier E. Effectiveness of the Pfizer-BioNTech and Oxford-AstraZeneca vaccines on covid-19 related symptoms, hospital admissions, and mortality in older adults in England: test negative case-control study. BMJ. (2021) 373:n1088. doi: 10.1136/bmj.n1088, PMID: 33985964 PMC8116636

[12] Huespe IA Ferraris A Lalueza A Valdez PR Peroni ML Cayetti LA. COVID-19 vaccines reduce mortality in hospitalized patients with oxygen requirements: differences between vaccine subtypes. A multicontinental cohort study. J Med Virol. (2023) 95:e28786. doi: 10.1002/jmv.28786, PMID: 37212340

[13] Kirsebom FCM Andrews N Stowe J Ramsay M Lopez BJ. Duration of protection of ancestral-strain monovalent vaccines and effectiveness of bivalent BA.1 boosters against COVID-19 hospitalisation in England: a test-negative case-control study. Lancet Infect Dis. (2023) 23:1235–43. doi: 10.1016/S1473-3099(23)00365-1, PMID: 37453440

[14] Zhao D Sun Y Li J Li X Ma Y Cao Z. Effectiveness of inactivated COVID-19 vaccines in preventing COVID-19-related hospitalization during the omicron BF.7-predominant epidemic wave in Beijing, China: a cohort study. BMC Infect Dis. (2024) 24:991. doi: 10.1186/s12879-024-09889-7, PMID: 39289630 PMC11406771

[15] Sarwar MU Waasia FZ Aloqbi AA Alandiyjany M Alqahtani RM Hafiz LA. Real-world effectiveness of the inactivated COVID-19 vaccines against variant of concerns: meta-analysis. J Infect Public Health. (2024) 17:245–53. doi: 10.1016/j.jiph.2023.12.005 38141544

[16] Centre for Health Protection. Updated reporting criteria of coronavirus disease 2019 (COVID-19), (2023). Available online at: https://www.chp.gov.hk/files/pdf/letters_to_doctors_20230126.pdf.

[17] Choi MH Wan EYF Wong ICK Chan EWY Chu WM Tam AR. Comparative effectiveness of combination therapy with nirmatrelvir–ritonavir and remdesivir versus monotherapy with remdesivir or nirmatrelvir–ritonavir in patients hospitalised with COVID-19: a target trial emulation study. Lancet Infect Dis. (2024) 24:1213–24. doi: 10.1016/S1473-3099(24)00353-0, PMID: 39025098

[18] Information Services Department. Booster Jab Plan Announced (2023). https://www.news.gov.hk/eng/2023/03/20230331/20230331_174122_804.html (Accessed 28 August 2024).

[19] Scientific Committee on Emerging and Zoonotic Disease and Scientific Committee on Vaccine Preventable Diseases. Consensus interim recommendations on the use of COVID-19 vaccines in Hong Kong (as of 1 august 2022), (2022). Available online at: https://www.chp.gov.hk/files/pdf/consensus_interim_recommendations_on_the_use_of_covid19_vaccines_in_hong_kong_1_aug.pdf (Accessed 20 August 2024).

[20] Hui P Yeung L Ko JK Lai TH Chan DM Chan DT. COVID-19 vaccination and transmission patterns among pregnant and postnatal women during the fifth wave of COVID-19 in a tertiary hospital in Hong Kong. Hong Kong Med J. (2024) 30:16–24. doi: 10.12809/hkmj2210249, PMID: 38226406

[21] Tsang NNY So HC Cowling BJ Leung GM Ip DKM. Effectiveness of BNT162b2 and CoronaVac COVID-19 vaccination against asymptomatic and symptomatic infection of SARS-CoV-2 omicron BA. 2 in Hong Kong: a prospective cohort study. Lancet Infect Dis. (2023) 23:421–34. doi: 10.1016/S1473-3099(22)00732-0 36521506 PMC9744442

[22] GISAID. VOC/VOI/VUM relative frequencies over time (2024). Available online at: https://gisaid.org/hcov-19-variants-dashboard/ (Accessed April 03, 2024).

[23] Tartof SY Slezak JM Puzniak L Hong V Frankland TB Ackerson BK. Effectiveness of BNT162b2 BA. 4/5 bivalent mRNA vaccine against a range of COVID-19 outcomes in a large health system in the USA: a test-negative case–control study. Lancet Respir Med. (2023) 11:1089–100. doi: 10.1016/S2213-2600(23)00306-5, PMID: 37898148

[24] Yan VKC Wan EYF Ye X Mok AHY Lai FTT Chui CSL. Effectiveness of BNT162b2 and CoronaVac vaccinations against mortality and severe complications after SARS-CoV-2 omicron BA.2 infection: a case–control study. Emerging Microbes Infection. (2022) 11:2304–14. doi: 10.1080/22221751.2022.2114854, PMID: 35980089 PMC9553171

[25] Tartof S Slezak J Puzniak L Hong V Xie F Ackerson B. Immunocompromise and durability of BNT162b2 vaccine against severe outcomes due to omicron and Delta variants. Lancet Respir Med. (2022) 10:e61–2. doi: 10.1016/S2213-2600(22)00170-9, PMID: 35533699 PMC9075856

[26] Nordström P Ballin M Nordström A. Risk of infection, hospitalisation, and death up to 9 months after a second dose of COVID-19 vaccine: a retrospective, total population cohort study in Sweden. Lancet. (2022) 399:814–23. doi: 10.1016/S0140-6736(22)00089-7, PMID: 35131043 PMC8816388

[27] Sternberg MR Johnson A King J Ali AR Linde L Awofeso AO. Application of a life table approach to assess duration of BNT162b2 vaccine-derived immunity by age using COVID-19 case surveillance data during the omicron variant period. PLoS One. (2023) 18:e0291678. doi: 10.1371/journal.pone.0291678, PMID: 37729332 PMC10511074

[28] Scientific Committee on Emerging and Zoonotic Diseases and Scientific Committee on Vaccine Preventable Diseases. Consensus interim recommendations on the use of COVID-19 vaccines in Hong Kong (as of 13 October 2022), (2022). Available online at: https://www.chp.gov.hk/files/pdf/consensus_interim_recommendations_on_the_use_of_covid19_vaccines_in_hong_kong_13_oct.pdf (Accessed 20 August 2024).

[29] Our World in Data. Coronavirus (COVID-19) vaccinations (2024). Available online at: https://ourworldindata.org/covid-vaccinations (Accessed July 03, 2024).

[30] The government of the Hong Kong Special Administrative Region. Government announces implementation arrangements for vaccine pass. (2022). Available online at: https://www.info.gov.hk/gia/general/202202/21/P2022022100781.htm (Accessed April 23, 2024).

[31] World Health Organization. XBB.1.16 Updated Risk Assessment, (2023). Available online at: https://www.who.int/docs/default-source/coronaviruse/05062023xbb.1.16.pdf?sfvrsn=f1845468_3 (Accessed 27 August 2024).

[32] World Health Organization. EG.5 updated Risk evaluation, (2023). Available online at: https://www.who.int/docs/default-source/coronaviruse/21112023_eg.5_ure.pdf?sfvrsn=35d6cf7d_1 (Accessed 27 August 2024).

[33] World Health Organization. JN.1 updated Risk evaluation (2024). Available online at: https://cdn.who.int/media/docs/default-source/documents/health-topics/sars/jn.1-9-february-2024.pdf?sfvrsn=9a39d825_3 (Accessed 27 August 2024).

[34] Scientific Committee on Emerging and Zoonotic Diseases and Scientific Committee on Vaccine Preventable Diseases. Consensus interim recommendations on the use of COVID-19 vaccines in persons with previous COVID-19 infection and children in Hong Kong (as of 12 march 2022), (2022). Available online at: https://www.chp.gov.hk/files/pdf/consensus_interim_recommendations_on_the_use_of_covid19_vaccines_in_persons_with_previous_covid19_infection_and_children_in_hong_kong_12mar.pdf (Accessed 20 August 2024).

[35] Centre for Health Protection. Factsheet on COVID-19 vaccination for persons with prior COVID-19 infection, (2024). Available online at: https://www.chp.gov.hk/files/pdf/factsheet_priorcovid19infection_eng.pdf (Accessed 20 August 2024).

[36] Tsang TK Sullivan SG Huang X Wang C Wang Y Nealon J. Prior infections and effectiveness of SARS-CoV-2 vaccine in test-negative studies: a systematic review and meta-analysis. Am J Epidemiol. (2024) 193:1868–81. doi: 10.1093/aje/kwae142, PMID: 38904437 PMC11637527

[37] HA Central Committee on Infectious Diseases and Emergency Response (CCIDER). Interim recommendation on clinical Management of Adult Cases with coronavirus disease 2019 (COVID-19). Hong Kong Special Administrative Region: Hospital Authority. (2023)

[38] World Health Organization. Interim statement on booster doses for COVID-19 vaccination: World Health Organization. (2021). Available online at: https://www.who.int/news/item/22-12-2021-interim-statement-on-booster-doses-for-covid-19-vaccination---update-22-december-2021 (Accessed April 03, 2024).

