# Supplementary material for: Using surveillance data to evaluate the effectiveness of inactivated/mRNA COVID-19 vaccine boosters in preventing fatal outcomes among severe COVID-19 cases during the current ambit of SARS-CoV-2 XBB and JN.1 variant circulation
Source: Front Public Health. 2025 May 14;13:1497399. doi: 10.3389/fpubh.2025.1497399 (PMC12116511; doi:10.3389/fpubh.2025.1497399)
Supplement: Supplementary file 1 [file Table_1.docx]

**SUPPLEMENTARY TABLES.**

Table 1. Full descriptive table of demographic characteristics between fatal cases and survived cases with severe/critical conditions (n=2,157)

|  | Total  (n=2,157) | Fatal cases  (n=764) | Survived cases  (n=1,393) | p-value |
| --- | --- | --- | --- | --- |
| **Age (mean ± SD)^**^** | 79.1 ± 11.5 | 83.4 ± 10.8 | 76.7 ± 11.2 | <0.001 |
| **Sex** |  |  |  | 0.923 |
| Female | 809 (37.5%) | 285 (37.3%) | 524 (37.6%) |  |
| Male | 1,348 (62.5%) | 479 (62.7%) | 869 (62.4%) |  |
| **Presence of chronic medical illness** | 1,873 (86.8%) | 676 (88.5%) | 1,197 (85.9%) | 0.107 |
| **Residing in RCHE^**^** | 411 (19.1%) | 198 (25.9%) | 213 (15.3%) | <0.001 |
| **Vaccination status^**^** |  |  |  | <0.001 |
| Unvaccinated/ incomplete with initial vaccination | 611 (28.3%) | 261 (34.2%) | 350 (25.1%) |  |
| Completed with initial vaccination | 1,029 (47.7%) | 334 (43.7%) | 695 (49.9%) |  |
| Completed with booster (inactivated) | 390 (18.1%) | 135 (17.6%) | 255 (18.3%) |  |
| Completed with booster (mRNA) | 127 (5.9%) | 34 (4.5%) | 93 (6.7%) |  |
| **Time between event and last valid dose^*^** |  |  |  | 0.001 |
| Unvaccinated | 439 (20.4%) | 187 (24.5%) | 252 (18.1%) |  |
| < 180 days | 351 (16.3%) | 126 (16.5%) | 225 (16.1%) |  |
| ≥ 180 days | 1,367 (63.3%) | 451 (59.0%) | 916 (65.8%) |  |
| **Reporting month** |  |  |  | 0.163 |
| Reported in 2023-01 | 22 (1.0%) | 11 (1.4%) | 11 (0.8%) |  |
| Reported in 2023-02 | 226 (10.5%) | 81 (10.6%) | 145 (10.4%) |  |
| Reported in 2023-03 | 67 (3.1%) | 17 (2.2%) | 50 (3.6%) |  |
| Reported in 2023-04 | 215 (10.0%) | 76 (9.9%) | 139 (10.0%) |  |
| Reported in 2023-05 | 685 (31.8%) | 235 (30.8%) | 450 (32.3%) |  |
| Reported in 2023-06 | 411 (19.1%) | 143 (18.7%) | 268 (19.2%) |  |
| Reported in 2023-07 | 154 (7.1%) | 68 (8.9%) | 86 (6.2%) |  |
| Reported in 2023-08 | 91 (4.2%) | 32 (4.2%) | 59 (4.2%) |  |
| Reported in 2023-09 | 96 (4.5%) | 31 (4.1%) | 65 (4.7%) |  |
| Reported in 2023-10 | 68 (3.2%) | 31 (4.1%) | 37 (2.7%) |  |
| Reported in 2023-11 | 23 (1.1%) | 10 (1.3%) | 13 (0.9%) |  |
| Reported in 2023-12 | 14 (0.6%) | 5 (0.7%) | 9 (0.6%) |  |
| Reported in 2024-01 | 85 (3.9%) | 24 (3.1%) | 61 (4.4%) |  |

^*^p-value <0.05; ^**^p-value<0.001 were found between fatal cases and survived cases with severe/critical conditions

Table 2. Univariate and multivariate logistic regression models of patients with different risk factor characteristics (n=2,157)

|  | Univariate | | Multivariate | |
| --- | --- | --- | --- | --- |
|  | OR (95% CI) | p-value | OR (95% CI) | p-value |
| **Age^**^**^++^ | 1.06 (1.05 – 1.07) | <0.001 | 1.06 (1.05 - 1.07) | <0.001 |
| **Sex**^+^ |  |  |  |  |
| Female | Referent |  | Referent |  |
| Male | 1.01 (0.84 – 1.22) | 0.890 | 1.36 (1.12 - 1.66) | 0.002 |
| **Presence of chronic medical illness** |  |  |  |  |
| No | Referent |  | Referent |  |
| Yes | 1.26 (0.96 – 1.65) | 0.094 | 0.90 (0.67 - 1.20) | 0.460 |
| **Residing in RCHE^**^**^+^ |  |  |  |  |
| No | Referent |  | Referent |  |
| Yes | 1.94 (1.56 – 2.41) | <0.001 | 1.51 (1.18 – 1.92) | 0.001 |
| **Vaccination status** |  |  |  |  |
| Unvaccinated or incomplete initial vaccination | Referent |  | Referent |  |
| Completed with initial vaccination**^**^**^+^ | 0.64 (0.52 – 0.79) | <0.001 | 0.70 (0.49 – 0.99) | 0.044 |
| Completed with booster (inactivated)^*+^ | 0.71 (0.55 – 0.92) | 0.010 | 0.63 (0.42 – 0.94) | 0.023 |
| Completed with booster (mRNA) **^**^**^+^ | 0.49 (0.32 – 0.74) | <0.001 | 0.57 (0.33 – 0.96) | 0.036 |
| **Time between event and last valid dose**  < 180 days | Referent |  | Referent |  |
| Unvaccinated | 1.33 (0.99 – 1.77) | 0.056 | 1.15 (0.73 – 1.82) | 0.550 |
| ≥ 180 days | 0.88 (0.69 – 1.13) | 0.300 | 1.10 (0.81 – 1.48) | 0.550 |
| **Reporting month** |  |  |  |  |
| Reported in 2023-01 | Ref. |  | Ref. |  |
| Reported in 2023-02 | 0.56 (0.23 – 1.36) | 0.190 | 0.68 (0.26 – 1.74) | 0.420 |
| Reported in 2023-03 | 0.34 (0.12 – 0.93) | 0.035 | 0.42 (0.14 – 1.20) | 0.100 |
| Reported in 2023-04 | 0.55 (0.22 – 1.33) | 0.180 | 0.71 (0.27 – 1.82) | 0.470 |
| Reported in 2023-05 | 0.52 (0.22 – 1.24) | 0.130 | 0.70 (0.28 – 1.76) | 0.450 |
| Reported in 2023-06 | 0.53 (0.22 – 1.28) | 0.150 | 0.71 (0.28 – 1.79) | 0.470 |
| Reported in 2023-07 | 0.79 (0.32 – 1.95) | 0.610 | 1.00 (0.38 – 2.60) | >0.990 |
| Reported in 2023-08 | 0.54 (0.21 – 1.40) | 0.200 | 0.63 (0.23 – 1.71) | 0.360 |
| Reported in 2023-09 | 0.48 (0.18 – 1.23) | 0.120 | 0.59 (0.21 – 1.61) | 0.300 |
| Reported in 2023-10 | 0.84 (0.32 – 2.21) | 0.720 | 1.10 (0.39 – 3.08) | 0.860 |
| Reported in 2023-11 | 0.77 (0.23 – 2.49) | 0.660 | 1.15 (0.33 – 4.02) | 0.820 |
| Reported in 2023-12 | 0.56 (0.13 – 2.16) | 0.400 | 0.98 (0.22 – 4.15) | 0.980 |
| Reported in 2024-01 | 0.39 (0.15 – 1.03) | 0.057 | 0.54 (0.19 – 1.51) | 0.240 |

Please refer to Appendix 2 for full table.

^*^p-value <0.05; ^**^ p-value<0.001 were found between within univariate logistic regression model

^+^p-value <0.05; ^++^ p-value<0.001 were found between within multivariate logistic regression model

Table 3. Simple descriptive comparison in vaccination status between study subjects and cases who firstly reported to CHP as fatal case

|  | Study subjects  (N=2,157) | Cases first reported to CHP as fatal case (N=2,098) | p-value |
| --- | --- | --- | --- |
| Age** | 79.1 ± 11.49 | 83.6 ± 10.97 | <0.001 |
| Sex** |  |  | <0.001 |
| Female | 812 (37.4%) | 983 (46.7%) |  |
| Male | 1,359 (62.6%) | 1,122 (53.3%) |  |
| Residing in RCH** |  |  | <0.001 |
| No | 1,746 (81.0%) | 1,363 (65.0%) |  |
| Yes | 411 (19.0%) | 735 (35.0%) |  |
| Vaccination status |  |  | 0.107 |
| Unvaccinated or incomplete with initial vaccination | 611 (28.3%) | 644 (30.7%) |  |
| Completed with initial vaccination | 1,029 (47.7%) | 1,012 (48.2%) |  |
| Completed with booster (inactivated) | 390 (18.1%) | 331 (15.8%) |  |
| Completed with booster(mRNA) | 127 (5.9%) | 111 (5.3%) |  |

^*^p-value <0.05; ^**^ p-value<0.001 were found between study subjects and cases who firstly reported to CHP as fatal case
